# Exploring the Relationship between Telomere Length and Cognitive Changes in Post-COVID-19 Subjects

**DOI:** 10.3390/biomedicines12102296

**Published:** 2024-10-10

**Authors:** Guillermo Efrén Villar-Juárez, Alma Delia Genis-Mendoza, J. Nicolas I. Martínez-López, Ana Fresan, Carlos Alfonso Tovilla-Zaráte, German Alberto Nolasco-Rosales, Ghandy Isidro Juárez-De la Cruz, David Ruiz Ramos, Mario Villar-Soto, Paola Mejía-Ortiz, Marlen Gómez Mendiola, Isela Esther Juárez-Rojop, Humberto Nicolini

**Affiliations:** 1 Escuela de Medicina, Universidad Anáhuac Querétaro, Querétaro 76246, Mexico; memovillar88@gmail.com; 2 Instituto Nacional de Medicina Genómica, Secretaría de Salud, Mexico City 14610, Mexico; adgenis@inmegen.gob.mx (A.D.G.-M.); paome.oz@gmail.com (P.M.-O.); 3 Hospital Psiquiátrico Infantil Dr. Juan N. Navarro, Servicios de Atención Psiquiátrica, Mexico City 14080, Mexico; gomezmendiola.marlenabigain@gmail.com; 4 Instituto Nacional de Psiquiatría Ramón de la Fuente Muñiz, Mexico City 14370, Mexico; drmaln@hotmail.com (J.N.I.M.-L.); fresan@imp.edu.mx (A.F.); 5 Divisón Académica Multidisciplinaria de Comalcalco, Universidad Juarez Autónoma de Tabasco, Comalcalco 86658, Mexico; alfonso_tovillaz@yahoo.com.mx; 6 División Académica de Ciencias de la Salud, Universidad Juarez Autónoma de Tabasco, Villahermosa 86100, Mexico; ganr_1277@live.com.mx (G.A.N.-R.); ghandyisidro@gmail.com (G.I.J.-D.l.C.); daruiz_914@hotmail.com (D.R.R.); 7 Hospital Regional de Alta Especialidad de Salud Mental, Villahermosa 86029, Mexico; mariovillarsoto@hotmail.com

**Keywords:** telomere length, cognitive changes, post-COVID-19, SARS-CoV-2

## Abstract

Background/Objectives: Emerging evidence suggests that patients suffering from COVID-19 may experience neurocognitive symptoms. Furthermore, other studies indicate a probable association between leukocyte telomere length (LTL) and neurocognitive changes in subjects with post-COVID-19 condition. Our study was designed to determine the correlation between telomere length and cognitive changes in post-COVID-19 subjects. Methods: This study included 256 subjects, categorized based on SARS-CoV-2 infection from 2020 to 2023. In addition, subjects with a psychiatric diagnosis were considered. Moreover, the MoCA and MMSE scales were applied. Telomere length was determined using a polymerase chain reaction, and statistical analysis was employed using ANOVA and X^2^ tests. Results: We identified a decrease in LTL in individuals with post-COVID-19 conditions compared to those without SARS-CoV-2 infection (*p* ≤ 0.05). However, no association was found between LTL and cognitive impairment in the subjects post-COVID-19. Conclusions: The findings suggest that LTL is affected by SARS-CoV-2 infection. Nonetheless, this important finding requires further research by monitoring neurological changes in subjects with post-COVID condition.

## 1. Introduction

The COVID-19 pandemic affected the mental and physical health of the world’s population. After the SARS-CoV-2 active infection was resolved, some symptoms remained in patients, and this condition is known as post-COVID-19 condition. Existing evidence shows that symptoms include dizziness, headache, fatigue, inattention, memory disorders, sleep disorders, anxiety, depression, obsessive compulsive disorder, paresthesia, altered consciousness, acute cerebrovascular disease, ataxia, seizures, neuropathic pain, impairment of taste, smell, or vision [1,2,3,4]. Recently, metabolic, functional, and structural alterations in the brains of post-COVID-19 patients have been related to changes in cognition and emotion processing. Likewise, Azcue et al. (2022) found a correlation between cognition, processing speed, abstraction capacity, visuospatial capacity, and decreased olfactory function in post-COVID-19 patients [5].

Telomeres are repetitive sequences at the ends of eukaryotic chromosomes, and they ensure genome integrity by preventing fusion between adjacent chromosomes [6]. The decrease in LTL in humans is related to the senescence process (biological aging). Existing evidence suggests that shorter TL and telomere dysfunction are associated with advancing age, as well as with obesity, smoking, type 2 diabetes, hypertension, chronic kidney disease, COPD, cardiovascular disease, and neurocognitive disorders [6,7].

Recent studies have shown that a shorter LTL is associated with a greater severity of COVID-19 [8,9,10]. Del Brutto et al. (2022) found that individuals with mild COVID-19 symptoms were at risk of developing late cognitive impairment compared to those without clinical and serological evidence of SARS-CoV-2 infection [11]. Scarabino et al. (2022) propose that telomere shortening is progressive in patients with cognitive impairment compared to a control group [12]. In contrast, Zhan et al. (2018) found that higher TL is associated with higher levels of cognitive ability [13]. Various studies have been consistent with these findings, and higher TL has been reported to be related to longer life expectancy [7,12].

The purpose of this research was to explore the association between telomere length and cognitive changes in post-COVID-19 subjects and individuals with psychiatric conditions.

## 2. Materials and Methods

### 2.1. Participants

We performed a transversal study. A total of 256 DNA samples were used: 142 samples from individuals with post-COVID-19 and 119 samples from subjects recruited before the COVID-19 pandemic. Both samples included subjects with diagnosed psychiatric conditions and individuals without psychiatric conditions. Patients with psychiatric conditions were recruited from the Hospital Regional de Alta Especialidad “Salud Mental Villahermosa”. The inclusion criteria for patients with psychiatric conditions included a previous diagnosis, age between 18 and 65, the provision of consent to participate, and the provision of a blood sample. The exclusion criteria included psychiatric conditions secondary to substance use. A psychiatrist evaluated all subjects. A total of 256 individuals (106 women and 148 men) were divided into the following four groups:

Group 1 comprised 75 individuals without SARS-CoV-2 infection but with a previous psychiatric diagnosis before the COVID-19 pandemic.

Group 2 comprised 62 patients with SARS-CoV-2 infection and at least one established psychiatric diagnosis.

Group 3 comprised 39 individuals without SARS-CoV-2 infection and without psychiatric conditions.

Group 4 comprised 80 individuals with SARS-CoV-2 infection but without diagnosed psychiatric conditions.

### 2.2. Study Design

COVID-19 diagnosis: To assess the impact of SARS-CoV-2 on telomere length, we used samples before the COVID-19 pandemic from previous studies conducted by our research team. From these samples, we included individuals with psychiatric conditions in Group 1 and those without such conditions in Group 3. The diagnosis of COVID-19 in subjects from Groups 2 and 4 was confirmed with a positive PCR test for SARS-CoV-2 before sample collection.

Psychiatric evaluation: The subjects in Groups 1 and 2 had psychiatric conditions, including mood and emotional disorders, neurodevelopmental disorders, neurodegenerative disorders, and disorders due to brain damage or dysfunction. They were evaluated and diagnosed by a psychiatrist according to the criteria of the Diagnostic and Statistical Manual of Mental Disorders (DSM-5) [14]. Group 4 included subjects who recovered from SARS-CoV-2 infection between August 2021 and December 2023. A face-to-face survey was administered to this group for the first time in 2021 (S1) and the second in 2023 (S2). Socio-demographic diagnostic scales (socio-demographic questionnaire, clinical history) and scales for assessing neurocognitive function (Mini-Mental State Examination, MMSE, and Montreal Cognitive Assessment, MoCA) were used. All participants signed an informed consent form for participation in this study.

### 2.3. Determination of LTL

Peripheral blood samples were collected from all study participants. These were collected in EDTA tubes and stored at −80 °C in a safe place in the INMEGEN psychiatric and neurodegenerative disease genomics laboratory. Subsequently, they were processed for DNA extraction from leukocytes using the Gentra purge kit from Qiagen (Hilden, Germany) and the determination of LTL [15].

### 2.4. Polymerase Chain Reaction (PCR) to Measure LTL

After DNA isolation, DNA quantity and quality were assessed by spectrophotometry (Nanodrop, 2000). A real-time polymerase chain reaction (rt-PCR) was performed to measure the average telomere length. Other studies have reported the primers used in this study [16]. The QuantStudio 6 Flex real-time PCR system (Thermo Fisher Scientific, Waltham, MA, USA) generated standard curves. LTL data are expressed using the 2^−ΔΔCT^ method, telomere threshold cycle (CT) values, and reference gene signals (ΔCT) [15]. We divided the LTL into four categories using quartiles: very short (0–0.0062 2^−ΔΔCT^), short (0.0062–0.0189 2^−ΔΔCT^), medium (0.0189–0.1075 2^−ΔΔCT^), and large (>0.1075 2^−ΔΔCT^).

### 2.5. Instruments

The scale used to assess cognitive function and determine the index of global cognitive function was the Mini-Mental State Examination (MMSE). The 35-item version was used, with scores above 24 indicating no cognitive impairment and below 24 representing probable cognitive impairment [17,18,19]. The Montreal Cognitive Assessment (MoCA) was the scale used to identify mild cognitive dysfunction; a score greater than 26 indicates normal cognitive function, and 1 point was added to patients with ≤12 years of schooling [19,20,21].

### 2.6. Analysis of Data

Data were expressed as interval variables (mean ± SD) and categorical variables (number, %), both at baseline (T0) and at follow-up (T1). The groups were analyzed using the chi-square test and the two groups using Student’s *t*-tests. Subjects with COVID-19 were included in a multivariate analysis of variance (MANOVA) model to evaluate changes in cognitive function between LTL classifications; partial eta squared (ƞ_p_^2^) was used for effect size comparison. Results were considered significant if *p* ≤ 0.05. All statistical analyses were performed using SPSS-26 and Prism (version 9.0) software.

## 3. Results

The mean age observed was 42.06 ± 11.67 years, and there was a greater frequency of men (148) (Table 1). Group 1 (29.3%) and Group 4 (31.3%) had a higher distribution of individuals. The most frequent psychiatric disorder was the diagnosis of schizophrenia, with 80 patients (Table 1). In this study, the mean LTL was 0.46 ± 1.28 (2^−ΔΔCT^). In addition, we found a relationship between LTL and age, showing that older individuals had shorter telomere length (*p* ≤ 0.05).

### 3.1. Relationship between LTL and Groups

Table 2 shows the means obtained for each group. We observed that the subjects without SARS-CoV-2 infection and with a psychiatric diagnosis (Group 1) had a higher LTL compared to all other participants (Group 2, Group 3, and Group 4) (1.052 ± 1.74). In contrast, the subjects without SARS-CoV-2 infection (Group 4) showed the shortest telomeric length compared to those in Groups 1, 2, and 3 (0.49 ± 0.65) (Table 2).

**Figure 1:**
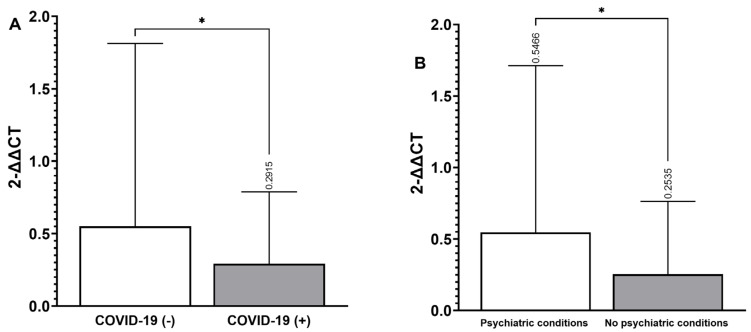
LTL in subjects with COVID-19 and psychiatric conditions. (**A**) Subjects with COVID-19 disease and without COVID-19. (**B**) Subjects with psychiatric conditions and without psychiatric conditions. Data are expressed by standard deviation; Student’s *t*-test was performed, * *p* ≤ 0.05. 2^−ΔΔCT^: 2-delta, threshold cycle.

Regarding the impact of SARS-CoV-2 infection on LTL, lower LTL was observed in individuals with COVID-19 compared to those without (*p* ≤ 0.05) (Figure 1A). In addition, persons who did not have a psychiatric disorder presented with a lower LTL than patients with a psychiatric condition (*p* ≤ 0.05) (Figure 1B).

### 3.2. Relationship between LTL and Cognitive Changes in Subjects with Post-COVID-19 Condition

Group 4 consisted of 52.5% male and 47.5% female participants, with a mean age of 43 ± 9.9 years. Participants in Group 4 showed cognitive changes on the MOCA and MMSE scales (62% and 27.5%, respectively). We found that cognitive changes persisted over time, as MOCA (<24) and MMSE (<26) scores were similar in both S1 and S2. We observed no association between LTL and cognitive changes in S1 and S2 using MOCA and MMSE (Table 3).

However, individuals with post-COVID-19 condition and medium LTL had a higher MMSE score in the second survey compared to the first survey (Table 4) (*p* = 0.023).

Interestingly, when we analyzed subjects with and without cognitive changes, according to LTL, we observed no association between LTL in the group with cognitive changes compared to the group without cognitive changes in post-COVID-19 subjects, as assessed by MOCA (*p* = 0.27 and 0.22) and MMSE (*p* = 0.22) (Table 5).

## 4. Discussion

In this study, shorter LTL was observed in post-COVID-19 individuals. Regarding the cognitive assessment, no association was found between LTL and changes in cognition in post-COVID-19 individuals. Previous research detected a lower average relative length of chromosomes in the peripheral blood leukocytes of COVID-19 patients compared to a group without COVID-19 [22]. Furthermore, it has been reported that COVID-19 patients may experience T cell lymphopenia, which is associated with telomere shortening and the severity of the disease [23,24,25,26,27]. Recently, a study revealed telomere shortening in sputum cells induced in healthcare workers with SARS-CoV-2 infection [28]. Similarly, a report suggests that COVID-19 severity accelerates the shortening of telomere length and deterioration [24]. In this context, a study has indicated that the shortening of T cell telomeres is associated with the immune response in individuals with viral infections (HIV, HBV, HCV, EBV, and CMV); this leads to extensive proliferation in T cells, resulting in telomere shortening, replicative impairment, and decreased immunocompetence [22]. Current investigations propose that individuals with short telomeres may have a suboptimal antiviral response to SARS-CoV-2 infection, potentially resulting in more severe and progressive COVID-19 disease [29]. In contrast, some authors indicate no correlation between TL and the severity of COVID-19 (e.g., involving invasive ventilation or death) in patients with SARS-CoV-2 infection [30,31].

We found no association between LTL and changes in cognition in post-COVID-19 individuals. Another study observed that longer LTL is associated with higher general cognition and improved performance in the cognitive domains of attention, speed, and executive function. In contrast, various researchers have reported a relationship between shorter LTL and cognitive impairment [32]. Campisi et al. (2024) show that healthcare workers with a shorter TL and accelerated biological aging have a decline in physical and cognitive functioning, affecting job performance [28]. In addition, shorter TL in nursing personnel who worked during the COVID-19 pandemic has been demonstrated, regardless of age group, suggesting that emotional exhaustion is associated with TL [33]. Our study found that individuals experienced cognitive exhaustion throughout the first post-COVID-19 year; however, it is not related to LTL. Previous research suggested that increasing serotonin levels boost the expression of telomerase reverse transcriptase (TERT) and telomerase activity through PI3K/Akt signaling [34]; this leads to the lengthening of telomeres and the induction of growth factors that promote neurogenesis and balanced mental health [34,35]. In addition, Grand et al. (2023) propose that if SARS-CoV-2 were to persist similarly to HCV, it could lead to severe and detrimental effects on the infected tissue, as well as genomic instability and DNA damage in individuals with psychiatric conditions [29]. Furthermore, a study suggests that TL is involved in the pathogenesis of age-related neurodegenerative diseases such as Alzheimer’s disease [36]. In contrast, a population-based MRI study found an association between longer LTL and larger brain and hippocampal volumes [37]. On the other hand, other studies propose an association between serotonin, TERT expression, and activation of the PI3K/Akt signaling pathway, indicating that treatment with antidepressants, lithium, and antipsychotics increases LTL [38,39]. Similarly, our findings showed that the group of patients with psychiatric conditions presented a greater length compared to the group without psychiatric conditions, possibly due to the use of antidepressants, anxiolytics, and antipsychotic medications [39] (Annex 1).

Our study observed that men had longer LTL than women. Some authors have suggested that TL is sex-specific, with girls having longer telomeres than boys from birth [40], and this relationship continues with age [41]. Furthermore, biological aging occurs due to molecular and cellular damage, as indicated by LTL and DNA methylation age (DNAmAge) [42]. Various reports demonstrate that telomere length is affected by oxidative stress, inflammatory processes, environmental factors, and lifestyle, psychosocial, and behavioral influences [43]. A recent report suggests that SARS-CoV-2 may accelerate epigenetic aging and is involved in developing post-COVID-19 conditions [44]. Also, one report suggests that blood leukocytes could be used as a biological sample to study the aging of the airways and lungs [28]. All these findings, taken together, propose that the shortening of LTL is a biological marker of stress-related alterations, participating in the physiological and genetic mechanisms of aging and various psychiatric disorders (e.g., depression, post-traumatic stress disorder, and chronic stress) [33,45].

Limitations. Our study presented some limitations. First, we noted a limited involvement of individuals with post-COVID-19 condition, resulting in a small sample size. Second, because of our study’s transversal design, we could not measure changes in telomere length and cognitive function. Third, we did not determine viral loads or viral genotypes, which could affect telomere length. Furthermore, our MANOVA analysis could not account for potential confounding variables because of the different diagnoses between individuals. Finally, we only had data on telomere length, so we could not determine other factors that may influence telomere shortening, such as telomerase activity, telomere dysfunction, and the cells’ *ability* to reverse telomere shortening.

## 5. Conclusions

We found no association between LTL and cognitive changes in individuals with post-COVID-19 condition; however, subjects with SARS-CoV-2 infection presented a shorter LTL. Interestingly, patients with psychiatric disorders may have larger LTL due to the use of antipsychotic, antidepressant, and anxiolytic medications. Based on these findings, future research should evaluate the long-term effects of SARS-CoV-2 on cellular health and aging, with the aim of improving the physical and mental health of individuals with post-COVID-19 condition.

## Figures and Tables

**Table 1:** Socio-demographic and clinical characteristics in subjects with psychiatric disorders with and without COVID-19.

Variable	F, Percentage, M ± S.D.	F, P
Age (years)	42.06 ± 11.67	
Sex		
Female	106, 41.4%	
Male	148, 57.8%	
Groups		
1	75, 29.3%	
2	39, 14.8%	
3	62, 24.2%	
4	80, 31.3%	
Diagnosis		
Mood and emotional disorders	12, 15%	
Neurodevelopmental disorders	17, 21%	
Neurodegenerative disorders	5, 6%	
Schizophrenia	80, 70%	
Telomere length (2^−ΔΔCT^)	0.46 ± 1.28	
Sex		
Female	0.58 ± 0.36	0.17
Male	1.58 ± 0.98	
Telomere length vs age (years)		
Very short	42.34 ± 11.15	2.671, 0.048 *
Short	45.22 ± 11.92	
Medium	41.05 ± 11.68	
Large	39.63 ± 11.31	

Note: numerical variables are expressed as mean and standard deviation (M and S.D.) for variables with a normal distribution. 2^−ΔΔCT^: 2-delta, threshold cycle, F: F-statistic, * *p* ≤ 0.05.

**Table 2:** Relationship between telomere length and subjects (Groups 1,2, 3, and 4).

LTL (2^−ΔΔCT^)	Group 1 M ± S.D.	Group 2 M ± S.D.	Group 3 M ± S. D	Group 4 M ± S. D	*p*
Very short	0.004 ±0. 001	0.005 ± 0.001	00.002 ± 0.001	0.003 ± 0.002	
Short	0.01 ± 0.005	0.023 ± 0.023	0.040 ± 0.027	0.012 ± 0.005	
Medium	0.062 ± 0.03	0.061 ± 0.030.	0.059 ± 0.023	0.064 ± 0.026	
Large	1.052 ± 1.74 *	0.6 ± 0.56	0.62 ± 0.79	0.49 ± 0.65	0.022 *

Note: numerical variables are expressed as mean and standard deviation (M and S.D.), the interquartile range for variables with a non-normal distribution. ANOVA test (*p* ≤ 0.05). 2^−ΔΔCT^: 2-delta, threshold cycle. * *p* ≤ 0.05.

**Table 3:** LTL and cognitive changes in subjects with COVID-19.

Assessment	LTL	MOCA	MMSE	F	df	*p*	ƞ_p_^2^
Survey 1				1.457	6	0.198	0.185
	Very short	23.20 ± 2.68	28.00 ± 4.30				
	Short	23.00 ± 2.98	29.90 ± 4.93				
	Medium	23.80 ± 1.92	33.40 ± 1.82				
	Large	25.75 ± 2.50	29.75 ± 1.50				
Survey 2				1.490	4	0.226	0.142
	Very short	26.67 ± 3.22	34.33 ± 1.16				
	Short	-	-				
	Medium	24.50 ± 3.92	30.30 ± 2.75				
	Large	25.78 ± 2.73	29.22 ± 4.12				

Note: the MMSE and MOCA scores are expressed as mean (M) and standard deviation (S.D.); MANOVA tests were performed. F: F-statistic, df: degrees of freedom, *p*: *p*-value, ƞ_p_^2^: partial eta squared.

**Table 4:** LTL and cognitive changes between surveys in subjects with post-COVID-19 condition.

Variable	Survey 1	Survey 2	P
MMSE vs. LTL			
Very short	34 ±1.41	27.66 ± 4.51	0.163
Short	-	-	-
Medium	29.37 ± 2.9	32.45 ± 2.46	0.023 *
Large	28.83 ± 036	29.66 ± 4.18	0.732679
MOCA vs. LTL			
Very short	22.25 ± 3.41	21.67 ± 4.16	0.37
Short	25.50 ±.71	25.50 ±.1.21	0.87
Medium	24.33 ± 3.05	22.45 ± 3.50	0.79
Large	23 ± 0.5	22.78 ± 4.38	0.22

Note: the MMSE and MOCA scores are expressed as mean (M) and standard deviation (S.D.); paired Student’s *t*-tests for each LTL category were used. * *p* ≤ 0.05

**Table 5:** Distribution of cognitive changes and telomere length in subjects with COVID-19.

	Cognitive Changes	No Cognitive Changes	X^2^, *p*
MMSE	6, 15%	34, 85%	
Very short	3, 50.0%	7, 20.6%	3.92, 0.27
Short	1, 16.7%	9, 26.5%	
Medium	0, 0.0%	10, 29.4%	
Large	2, 33.3%	8, 23.5%	
MoCA	29, 72.5%	11, 27.5%	
Very short	9, 31.0%	1, 9.1%	4.39, 0.2
Short	5, 17.2%	5, 45.5%	
Medium	7, 24.1%,	3, 27.3%	
Large	8, 27.6%	2, 18.2%	

Note: cognitive changes and LTL categories are expressed as n (%); the chi-square test was used. χ^2^: chi-square statistic, *p* ≤ 0.05.

## Data Availability

Data are contained within the article.
